# Pathological Anatomy

**Published:** 1837-01-01

**Authors:** 


### PATHOLOGICAL ANATOMY.

I. LECTURES ON THE MORBID ANATOMY OF THE SEROUS & MUCOUS MEMBRANES. In Two Volumes. By *Thomas Hodgkin*, M.D. Demonstrator of Morbid Anatomy, and Curator of the Museum at Guy's Hospital, &c. &c. &c. Vol. I. On the Serous Membranes, and as appended subjects, Parasitical Animals, Malignant Adventitious Structures and the Indications afforded by Colour. 8vo. pp. 402. London, 1836.

II. THE CLINIQUE MEDICALE. By *G. Andral.* Condensed and Translated by *D. Spillan*, M.D. &c. Part V. Containing the Diseases of the Abdomen.

IT is unnecessary for *us* to say any thing on the value of the knowledge of morbid anatomy, nor need we dwell on the best means of becoming acquainted with its facts. Those means are within the reach of the surgeon or physician who practises in cities, but his less fortunate brethren in the country must be content to derive second-hand from books, what we obtain, fresh and fresh, from the dead body.

For the wide diffusion of information, the periodical press is chiefly to be thanked. Its powers must be great, and its objects should be good. Those who wield the former incur a serious responsibility, and without indulging in a mawkish affectation, we may say that a conscientious critic must feel that his duty to the public is a high one. For our own parts, we have endeavoured to encourage to the utmost the study of facts, to repress as far as possible the tendency to speculation, and whilst we have been far from prejudiced in favour of things that are merely recommended by antiquity, we have not taken the Curtian leap that some would appear so much inclined to do, into the abyss of novelty and of extravagance. To pursue so eclectic a course without error, would imply such an amount of judgment and sagacity, as we cannot pretend to lay claim to. But our faults, whatever they may be, have been mostly we hope on the right side, and if we have sometimes withheld, in the first instance, our belief in facts or in doctrines which have subsequently proved correct, the refusal has originated in the strict application of those rules of evidence, which are too often disregarded by writers and by reasoners on medicine.

None of our contemporaries have exceeded us in the earnestness with which we have insisted on the cultivation of morbid anatomy, nor in the zeal with which we have long endeavoured to disseminate its facts. We are not of that ultra montane school, whose researches begin and end in the dead-house, and who only look upon the living as the raw material of the dead. But we are fully convinced of the necessity of basing both the science and the practice of medicine on pathological facts, and we are convinced also that we have not yet attained as accurate a knowledge of those facts as is possible. We have not allowed a number of this Journal to be published for these two or three last years, without at least one article expressly dedicated to the subject of morbid anatomy. That plan we shall pursue, and we shall endeavour to give more completeness to its details; and we trust that we shall put our readers in possession of the current state of pathological information.

The principal works on morbid anatomy that have appeared within the quarter, are the two whose titles introduced these observations. M. Andral's work is, as its name implies, of a clinical character, yet it is so grafted on anatomical researches, and embodies so fully the facts which such researches elicit, that it cannot be separated from writings more ostensibly pathological.

Dr. Hodgkin is well known as a zealous morbid anatomist. His situation at Guy's Hospital gave him opportunities which his talents and his zeal have seized. His knowledge was conveyed to the pupils of the hospital in the form of lectures, and from the pupils it is offered to the public, through the medium of the press. The volume before us is the first of a series, probably three or four in number, and, judging from this, we may confidently prognosticate that all will be acceptable to the profession.

The subjects treated, are, as may be gathered from the title-page, the lesions of the serous membranes, parasitical animals, malignant adventitious structures, and the indications afforded by colour. Their examination occupies twelve lectures, which constitute so many chapters. The first is, of course, an introductory one—the second is on the serous membranes in general—the third is on the arachnoid—the fourth on the pericardium—the fifth on the pleura—the sixth on the peritoneum, tunica vaginalis, and bursæ—the seventh is crowded with parasitical animals—the eighth is on adventitious serous membranes—the ninth on the general characters of malignant disease—the tenth is on scirrhous and on fungoid tumors—the eleventh is on the colours of animal tissues—the twelfth reverts to malignant disease in general.

Although the present notice is in the body of this number of our Journal, the work itself was published too late in the quarter to enable us to do more than snatch a small niche for it. If we can give a sketch of the first and second lectures, that is the utmost we may hope to do.

The introductory lecture points out the utility and the necessity of an acquaintance with morbid anatomy. It gives a slight history of the progress of the science. The Greeks, the Romans, and the Saracens, were rather occupied with the disputes of the empirical and dogmatic sects, than engaged in the examination of the dead, which, indeed, either the religion or the prejudices of the period forbad. It does not, in fact, appear, says our author, that any distinct treatise had been written on the subject of *morbid anatomy* until the year 1674, when Thomas Bartholin, a Dane, published a work, entitled *Consilium de Anatomiâ Practicâ, ex Cadaveribus Morbosis adornandâ.* This was quickly followed by the celebrated *Sepulchretum, sive Anatomia Practica*, of Theophilus Bonetus, a physician of Geneva; respecting whom it has been said, "egregie, si quis alius, meritus est." He has given us, in an anatomical order, the result of his own careful observations, during forty years; together with a laborious collection from the works of his predecessors and contemporaries.

Morgagni, the father of morbid anatomy, appeared. After him came Lieutaud, Ludwig, who published in 1783, and Sandifort, who followed hard upon him. The morbid anatomy of Dr. Baillie did much for himself, and a great deal for medicine. It was not so minute as modern morbid anatomy is; but could that be expected? A consideration of the causes which have done so much for morbid anatomy of late years, must answer that question in the negative. Those causes are very properly considered by Dr. Hodgkin to be chiefly two.

"The first of these was the special cultivation of *General Anatomy*, the outlines of which were faintly sketched by Dr. Carmichael Smith. The higher praise, however, is due to the great Bichat; who, while he seems to have possessed the merit of originality equally with Dr. Smith, has pursued the subject into its minutest details; so as to leave little to his successors, but the introduction of a few slight modifications and divisions.

This indirect, but very important, service to the cause of pathological anatomy was not the only one which it received from Bichat. One of his last labours was the delivery of a course of lectures on *Morbid* Anatomy, of which he made his *General Anatomy* the basis. On the same basis, Pinel laid the outlines of his *Nosographie Philosophique;* and the numerous individuals of superior talent, who were fortunately brought together in the Clinical School of Corvisart—amongst whom Bayle, Laennec, and Rostan must be specially noticed—following up the same plan, have made most important advances in this department." 12.

The second cause is the establishment of the 'doctrine physiologique.' By morbid anatomy it was reared—by more minute morbid anatomy it has been overthrown—and both its supporters and opponents cultivated, with the ardour of enthusiasm and of party, the science from which they expected the elements of victory.

After paying a well-merited tribute of respect to Louis, and pointing him out as the originator of the statistic method, Dr. H. proceeds to indicate the order he intends to follow in his observations, the arrangement, in fact, of his "course." The basis of that arrangement is general anatomy, and he does with a diseased tissue what the healthy anatomist does with a sound one—investigates its physical states, its composition, and its functions. His method of proceeding may be gathered from the following table, which sets out the deviations from the natural state, that an unsound organ may exhibit.

DEVIATIONS FROM THE NORMAL STATE; CONSISTING,

1. In deficiency.
    *a* The result of suspended development.
    *b* Loss sustained.
2. In excess.
3. In form.
4. In appearances which may be regarded as the result of ordinary inflammation.
5. In appearances which are the result of scrofula.
6. In appearances which are the result of diseases called malignant, or resembling them in structure.
7. In hydatids in the particular organ.
8. In the effects of accidental injury.

Dr. Hodgkin proceeds to develop, seriatim, each head of the preceding arrangement. That is quite unnecessary, as our readers may see at a glance its characters and objects.

The second lecture is devoted to the morbid anatomy of the serous membranes in general. Our author deems it necessary to explain why he commences with them, rather than with the cellular tissue, which is a common element of all membranes, of all organs, and of all tissues. His reasons are unsatisfactory; but it is a matter of so little consequence, that we dismiss it. Provided all tissues are investigated, it signifies little, perhaps, with which we start.

The general anatomy of the serous membranes first attracts the attention of our author. The only remarks on this head that deserve notice, are some observations on the ultimate structure of the tissue, founded on its examination by Mr. Lister, the possessor of a well-known powerful microscope. Our readers are probably aware of the discrepancies that have always characterized the results of microscopic researches. The last microscopist has usually contended that his predecessors were deceived by optical illusions, and the possessor of the newest instrument has fondly hoped that *that* will decide the controversy. Whether we have really arrived at that perfection in the construction of the microscope, which will enable us to form definitive and correct opinions on the ultimate structure of the tissues submitted to it, or whether that structure is too subtle to be ever fairly cognizable by even the assisted eye of sense, are questions that it is at present impossible to determine. We shall simply state what Mr. Lister and Dr. Hodgkin have seen.

"The ultimate structure of these membranes, like that of the cellular tissue, has been regarded by most minute observers, both British and foreign, as consisting of extremely minute fibrillæ, so combined as to form lamellæ. By many modern physiologists, French and German, as well as English, these fibrillæ are believed to be composed, in their ultimate analysis, of globules, arranged like strings of beads. This opinion, I am fully convinced, has its sole foundation in an optical illusion. My friend Joseph Lister, who has carried the powers of the microscope far beyond any thing to which they had previously attained, has very minutely examined this, as well as most of the other animal tissues. He and myself have spent hours in the most careful examination of the subject, and not the smallest doubt is left on our minds as to the absolute fallacy of the globular theory. In fact, this theory appears to be completely set aside by *à priori* reasoning. The extremely delicate textures of which animals of the most minute microscopic size are formed precludes the possibility of the ultimate structure being visible to our eyes, however aided by microscopes. There are animalcules of such minute size, that their entire bulk is not equal in volume to that ascribed by the advocates of the globular theory to one of their integrant or primitive molecules. Lucretius has well observed, concerning the ultimate particles of matter, *Nequeunt oculis rerum primordia cerni.* The perfectly-formed membranes appear, I believe without exception, to consist of fibrillæ of tolerably even size, and in no respect resembling a chaplet of beads. Meckel denies the existence of the fibrous structure in the serous and cellular membranes; but believes that they consist of a coherent, homogeneous substance, which is viscid, amorphous, and, in the cellular membrane, scarcely solid; but more condensed, and coagulated in broad layers, in the serous. I imagine that Meckel must have been led to form this opinion from his having principally examined recent and imperfectly-formed membranes, to which the descriptive character given by him pretty accurately applies." 27.

Dr. Hodgkin insists on the approximation in the character of their secretions, of the serous to the mucous membranes, or rather of the convertibility of the one into the other. Our excellent friend is ingenious and argumentative, but perhaps the affinity is pushed rather hard. Yet, as he remarks, the synovial membranes and the vaginal sheaths do form a sort of half-way house between the pure serous membranes, as the arachnoid, and the mucous. Let us proceed to the lesions of the former.

The alterations in their secretion are noticed first, These are, an absence of secretion, when the serous sac is unnaturally dry—a rare affection ; the production of gas, which, if possibly a secretion, is in most cases either of extraneous origin, as when the intestine opens into the peritoneum, or a vomica into the pleura : or the result of decomposition ; increase of the natural secretion, constituting hydrocephalus, hydrothorax, &c.; perversion of the secretion, which may assume a mucous character, or a bilious, or a bloody; on the latter point, we shall extract a remark or two of Dr. Hodgkin's, because it is frequently a source of some difficulty, if not of error, to the inexperienced practitioner.

"The admixture of blood is as often a cadaveric, as a morbid phenomenon. To make an accurate distinction between these two forms, we must, as in the case of the presence of gases, be guided by the nature of the accessary circumstances. If the patient have died suddenly, and the blood have universally retained its fluidity—if the various tissues are soft and flabby, and have become stained by the transudation of the bile or the blood—there can be little doubt of the sanguinolent colour of the serum in the different cavities being a cadaveric appearance, except when the patient is known to have been labouring under scorbutus, in which case both causes have probably concurred. When we find the sanguinolent secretion confined to a particular cavity—when the membrane containing it appears to be rather injected than stained—and, more decidedly, when a local determination or injury is known to have taken place at the part—we may infer that the appearance of blood is morbid, rather than cadaveric. After all, I must confess that I have seen many cases in which it was very difficult, if not impossible, to draw the line." 33.

To proceed. Blood has been found in the pericardium, without appreciable lesion : and in the pleura : the fluid of ascites is at times sanguinolent: so is that of hydrocele, which may be superseded by pure blood, and become hæmatocele. Chyle and milk have been said to have been found in the peritoneum, the former just possible, the latter not; in such marvellous cases, the fluid has been probably a light-coloured, puriform secretion.

The lesions of the membranes themselves are :—

1. Deficiency. The arachnoid and even the pleuræ may be wanting in cases of monstrous fœtus. The pericardium has undoubtedly been absent. The peritoneum may be open in front. When the testis does not descend, its prolongation is, of course, not present.

2. Excess. Protrusions of the viscera they contain, cysts, &c. furnish examples of this.

3. The effects of inflammation. Of these a good account is given, taken from Villermé and Dupuytren, their researches are probably familiar, and have been published at various times and in various forms in this country. We shall, therefore, confine ourselves to an allusion to one or two particulars, and to the mention of the points on which our author differs from those gentlemen.

*a.* False membranes are the product of inflammation of the serous tissue, and those false membranes are themselves subsequently organized. The time at which vessels begin to be visible in false membranes appears to be very different in different cases. Villermé has never seen them before the twenty-first day, from the invasion of the malady; but Stoll mentions cases in which organization appeared to have taken place as early as the twelfth, or even the eighth day. Our author thinks that it has been witnessed earlier.

*b.* Villermé and Dupuytren conceive that the organizable matter is what they term concrete pus. This is first effused in the form of villi, attached to the serous surface that forms them. Being organized, this substance is absorbed, and cellular tissue deposited finally in lieu of it. Our author joins issue with Villermé on the nature of the original secretion, which he does not think to be either pus or puriform, but plastic lymph, the result of coagulation, the coagulable lymph, in short, of John Hunter. It may be observed, that Villermé and his associates, by employing the term concrete pus, imply a difference between it and common pus, though their subsequent remarks in many points obscure the distinction. Premising these remarks, which are requisite for the right understanding of our author's argument, we subjoin the latter.

"I find it impossible to unite with these authors, and with Sir Everard Home, Laennec, and several others, as to the organizable properties of pus or puriform secretions. I believe them to be always, more or less, excrementitious; and that where an outlet from the body is not afforded, they invariably retard cure, by interfering with the progress of organization in those substances which are formed in conjunction with them, and are susceptible of this change. You will remember, that I have mentioned several varieties in the effusions met with in the cavities of serous membranes; and I would particularly recall your attention to one form which has the power of coagulating like blood. This I believe to be the most eminently plastic effusion, which, as coagulation advances, very rapidly assumes the form of tender diaphanous films, which we find separated by, and infiltrated with, a limpid, but often straw-coloured serum. They are not in general originally applied and attached to the surface of the inflamed serous membrane; and it frequently happens that the union is ultimately only partial, yet amply sufficient for their organization and support. Though these diaphanous films, or incipient false membranes, do not appear to be necessarily formed in immediate contact with the surface of the serous membrane, it is no less obvious, that their formation is often, if not generally, materially influenced and modified by the membranes in the cavities of which they are contained, as may be best seen in the peritoneum; since we find them assuming the form, and adapt themselves to the irregularities of those cavities, even where they are accompanied by a large proportion of serum.

The opinion which I wish to support in opposion to Villermé and which I have been led to form by a careful examination undertaken in consequence of perusing the work of Villermé, is, I believe, in some points, little more than a return to the doctrine of John Hunter, though not borrowed from him. I find myself perfectly in accordance with this great master of his profession, in regarding the formation of the tender diaphanous films composed of the plastic part of the effusion (the coagulable lymph of John Hunter) as the result of a process of the same nature with the coagulation of the blood. Hence, from the first, they are continuous and homogeneous, even whilst they can scarcely be said to be solid; a state which they attain by the separation of the serum, rather than by the aggregation of floating particles or villi." 45.

The theory of coagulable lymph seems in many respects more consistent with reasoning and indeed with facts, than that of "concrete pus."

Whether, however, the deposition of the lymph can be deemed, in all cases, a species of coagulation may perhaps be considered problematical.

The mode in which the vessels penetrate, or are developed in the false membrane, has long been a fertile source of speculation. Are they developed in the new nidus, or are they prolongations of the vessels of the membrane on the surface of which the false membrane has been deposited? When we look at the growth of bone, a tissue which illustrates in a remarkable degree the phenomena of growth, we find vessels carrying *red* blood in the centre of the cartilage, when a continuity of such vessels, with those of contiguous tissues, can be with difficulty, if at all, demonstrated. But all the concurrent phenomena seem to shew that those vessels are the productions of others, and that the neighbouring membranes, periosteum, perichondrium, the cellular, or the synovial, as the case may be, are the real sources of supply. To be sure, these investigations relate only to the deposit of the earthy material, and the earlier phenomena of growth are less certain. In the soft and almost mucous tissues of the embryo, it is far from clear how the vessels are developed, and the exact operation of the nisus formativus is a subtle mystery, and will probably continue so. The phenomena of inflammation and of growth are so allied, the formation of new tissues and the natural development of the old are so analogous, that we turn from the one to the other for assistance in the comprehension of their several processes.

To return to the organization of false membranes. It is more natural to conclude that their vessels do originate in the vessels of the parent tissue. Dr. Hogdkin's opinion, or rather theory, is this:—

"That, at the inflamed part, the minute blood-vessels not merely become distended, but that their delicate parietes, and the structure through which they ramify, become softened, and, yielding to the pressure of the blood in the distended vessels, give way at numerous minute points. This I believe to be the explanation of the fact, that when we raise an adherent false membrane of a few days standing, we find that the original serous membrane beneath has lost somewhat of its polish, and presents numerous minute bloody points. The very small quantity of blood thus escaping from its vessels does not diffuse itself, but is received into the soft substance of the false membrane, which accordingly exhibits numerous bloody points on the surface which has been detached from the serous membrane. These spots, which are at first irregular, afterwards have a dendritic appearance; and extending in length, they become vessels. These vessels being feebly supported, are distended and larger than those in the original structure from which they proceed; hence the redness of newly-organized false membranes. At a subsequent period, these vessels contract, and may become nearly or quite visible." 50.

We must confess that this hypothesis does not greatly help us. *Why* should minute extravasations of blood precede the formation of the vessel—*how* does that extravasation become a vessel? In reference to the first question, we may observe that there appears to be no necessity whatever for such effusion; the plastic lymph, offering quite as good a raw material for bloodvessels, as the coagulum. In reference to the second, we may remark that although our author expresses his inclination to believe the fact, he is not so indiscreet as to attempt an explanation. We fear that this is one of the many ultimate phenomena, on which the utmost information that we can anticipate obtaining, amounts only to probability.

There is an important law of inflammation of serous membranes, the interest of which may justify our alluding to it. At the same time that it is inclined to spread, it displays after injury, a tendency to limitation, particularly if the opposite points of two serous surfaces are wounded. The following illustrations of this fact are striking.

"If a patient has recovered from a penetrating wound of the thorax, by which both the pleura costalis and a corresponding spot of the pleura pulmonalis have been injured, you will very probably find the pleuritic adhesion confined to the immediate neighbourhood of the wound. Bichat had an opportunity of examining the body of an individual who had been affected with melancholy, under the influence of which he had, at different periods of his life, wounded himself with a knife twelve or fifteen times. In the most recent wounds, the intestines adhered to the parietes; in those which were somewhat older, they were connected by bridles; in a third class, which were of longer standing, the bridles were longer, and more slender; and in those which were the oldest, and of very long standing, the connexion was dissolved. You must, all of you, be aware of the very great danger which attends a wound of the dura mater; which, producing an opening into the cavity of the arachnoid, is extremely liable to lead to acute inflammation of that membrane, seldom affecting less than the entire surface of one hemisphere, and often extending over both. Yet, if the injury have been still greater, and have affected not only the arachnoid lining the dura mater, but that which covers the brain, and have even produced the loss of a portion of that organ itself, it is by no means uncommon for the symptoms to be comparatively slight. The late Henry Cline, jun. used to lay considerable stress upon this fact, in conjunction with injuries of the head; and suggested the propriety of designedly lacerating the arachnoid and pia mater, in cases in which the dura mater alone has been wounded." 55.

Perhaps the latter recommendation is going a little too far. Did we follow Mr. Cline's advice, we should perhaps be only leaping out of the frying-pan into the fire. We *might* increase the tendency to limitation of the inflammation—we *should* increase the risk of hernia cerebri.

False membranes gradually display a diminution of their vascularity, and become pale, and even indurated. They may even assume a cartilaginous, nay, ossific character, the circumstances favouring or determining which are dubious. We have reason to believe that the following observation of our author's is well founded; at least, it coincides with facts that have come under our own observation.

"The contraction of the organized product of inflammation, to which I have already alluded, often gives rise to strange deformity of the parts to which they are united. Laennec was the first who pointed out the remarkable retrocession of one side of the chest which takes place when a patient survives and recovers from empyema. This he ascribed to the absorption of the fluid; but, as I suggested to the Physical Society of this Hospital, in a Paper which I read on the application of the stethoscope, in the year 1822, I believe the true cause to be the contraction to which I have just alluded. I am confirmed in this opinion from having observed similar effects, where the cause, to which Laennec refers, could not have operated." 57.

The best marked case of contraction of one side of the thorax that we ever witnessed, was the consequence of encephaloid growths in the lung, independently of any pleural effusion at all.

The subserous cellular tissue is often the seat of inflammation and of effusion. Its proneness to the latter is, or seems to be, in the ratio of its looseness. Serous infiltrations are very frequent in the sub-arachnoid cellular tissue. We doubt, however, the absolute correctness of this axiom. Other circumstances exert an influence. Beneath the sub-arachnoid cellular tissue is the pia mater, a very vascular membrane; there effusions are common. The cellular tissue beneath the occipito-frontalis muscle is very loose, much looser than the subcutaneous cellular membrane. Yet inflammatory effusions are much more frequent in the latter than in the former, because the causes of inflammation are more rife in it. When inflammation *does* invade a loose cellular tissue, effusion runs along it like wildfire, because there is nothing to arrest it; but, accidental circumstances apart, it is doubtful whether that cellular membrane is particularly *prone* to inflammation.

Gangrene of a serous membrane is rare. Lieutaud has mentioned one instance in the pericardium; and the highly offensive and discoloured condition of effusions into some of the serous cavities, described by Bichat, was probably attended with a state of membrane approaching to sphacelus. Dr. H. has never witnessed an example of it, unless the gangrene was one of extension from neighbouring parts.

The effects of struma on the serous membranes are not confined to scrofulous deposits in the cellular membrane, or on the attached surface of the serous. Both *in* the latter and *on* it, we see collections of tuberculous or scrofulous matter. In them we see also miliary tubercles.

Malignant disease is usually confined to the sub-serous cellular tissue. Yet minute bodies, having the character of scirrhus, have been found in the substance of the serous membranes, in individuals who had malignant disease elsewhere.

Serous membranes, being membranes of isolation, do not usually transmit inflammation to each other. But a sympathy unites them when remote. Thus the pericardium is apt to inflame, in company with the synovial membranes; or the pleura, or even the arachnoid, may do so.

Such is a sketch of the principal portions of the first two lectures of our able and amiable author. In our next number we shall continue the subject, and present a lengthened notice of the pathological facts contained both in this and in other works. We recommend the present volume both to the young student and the old.

